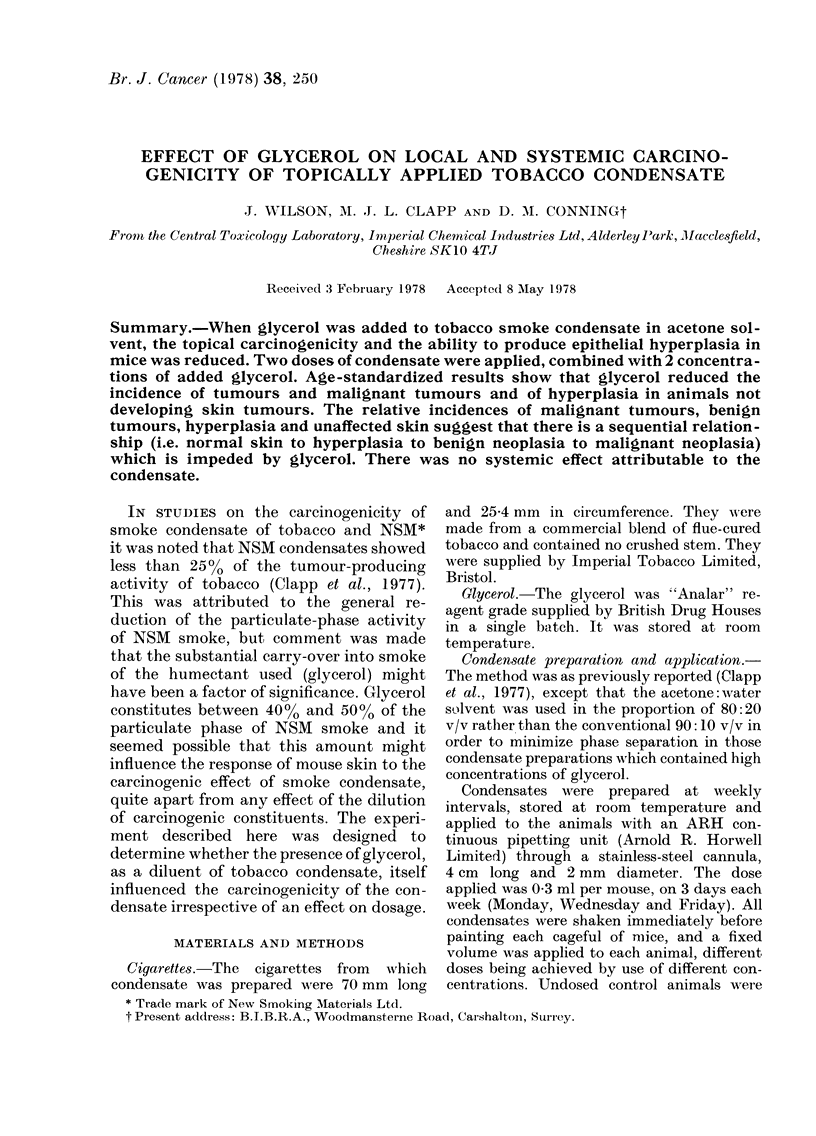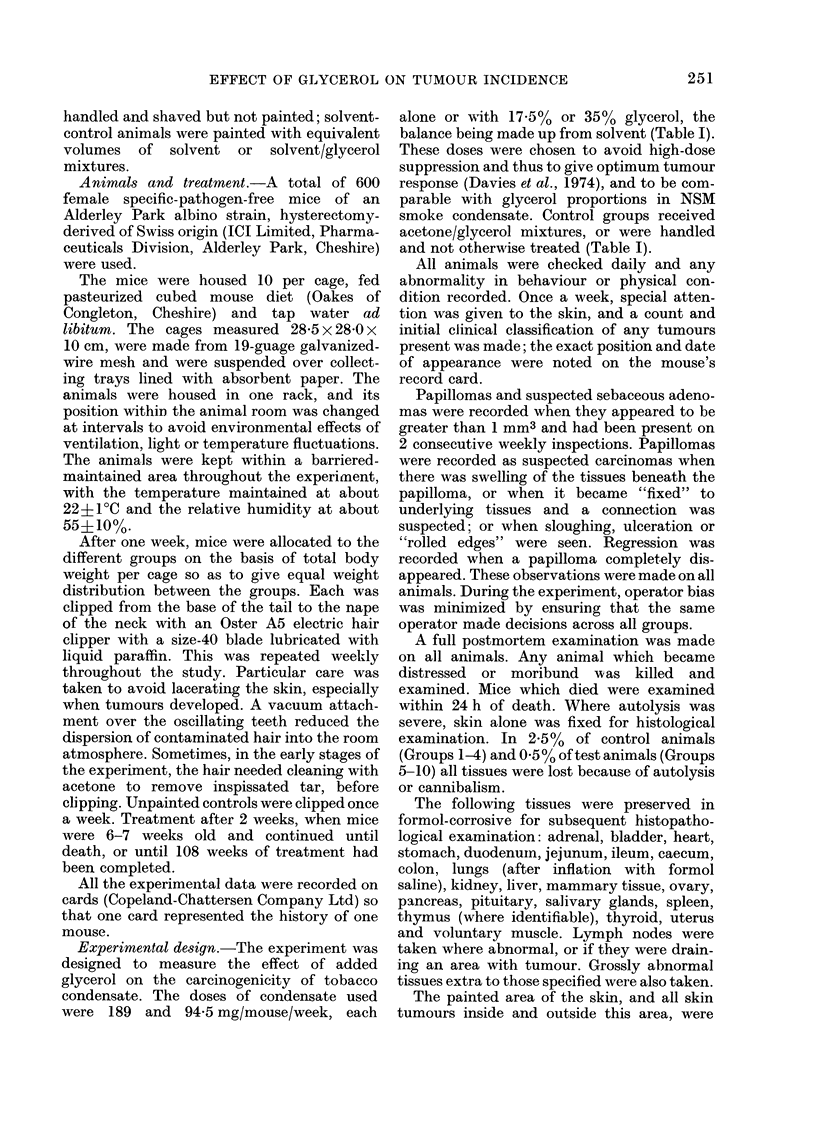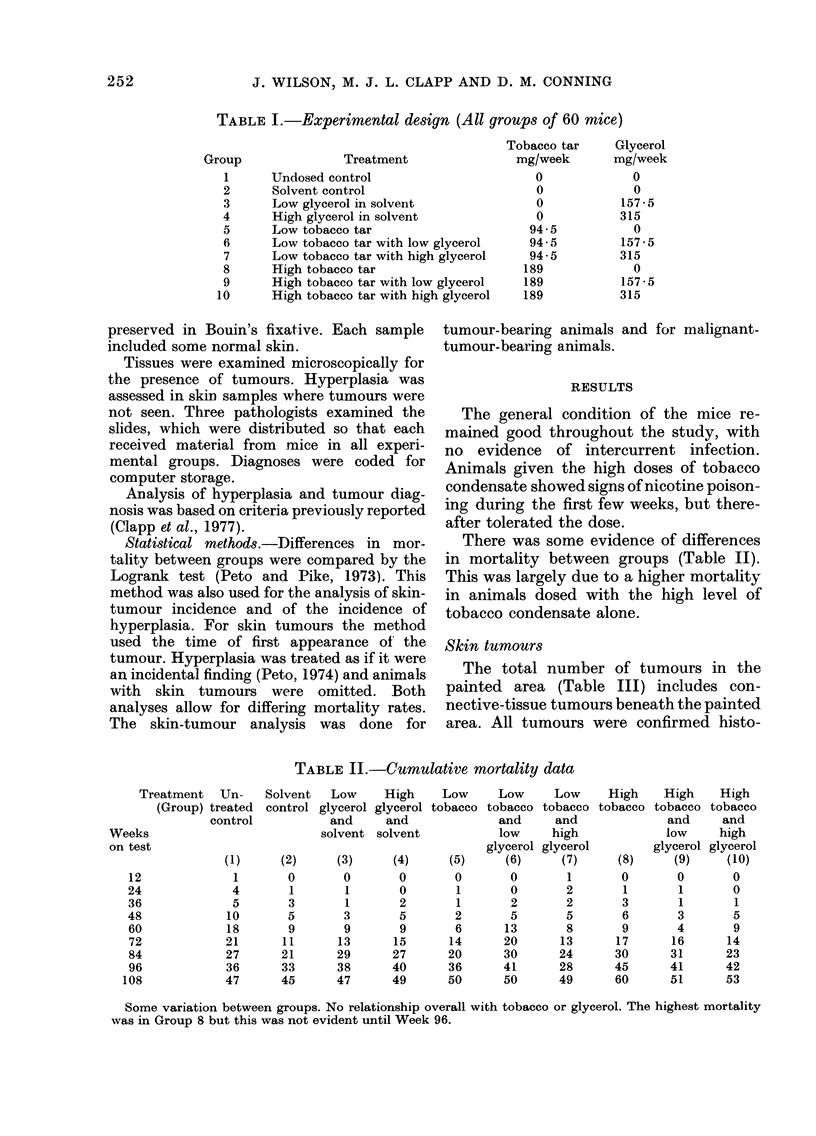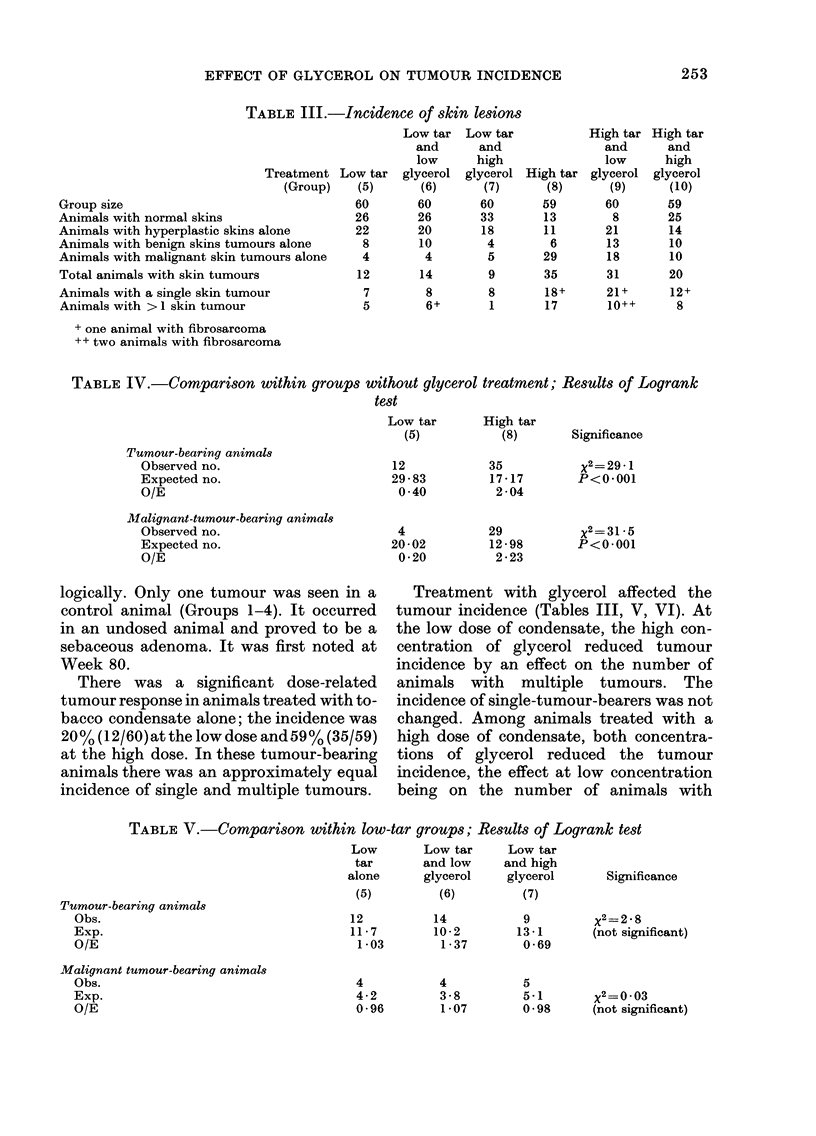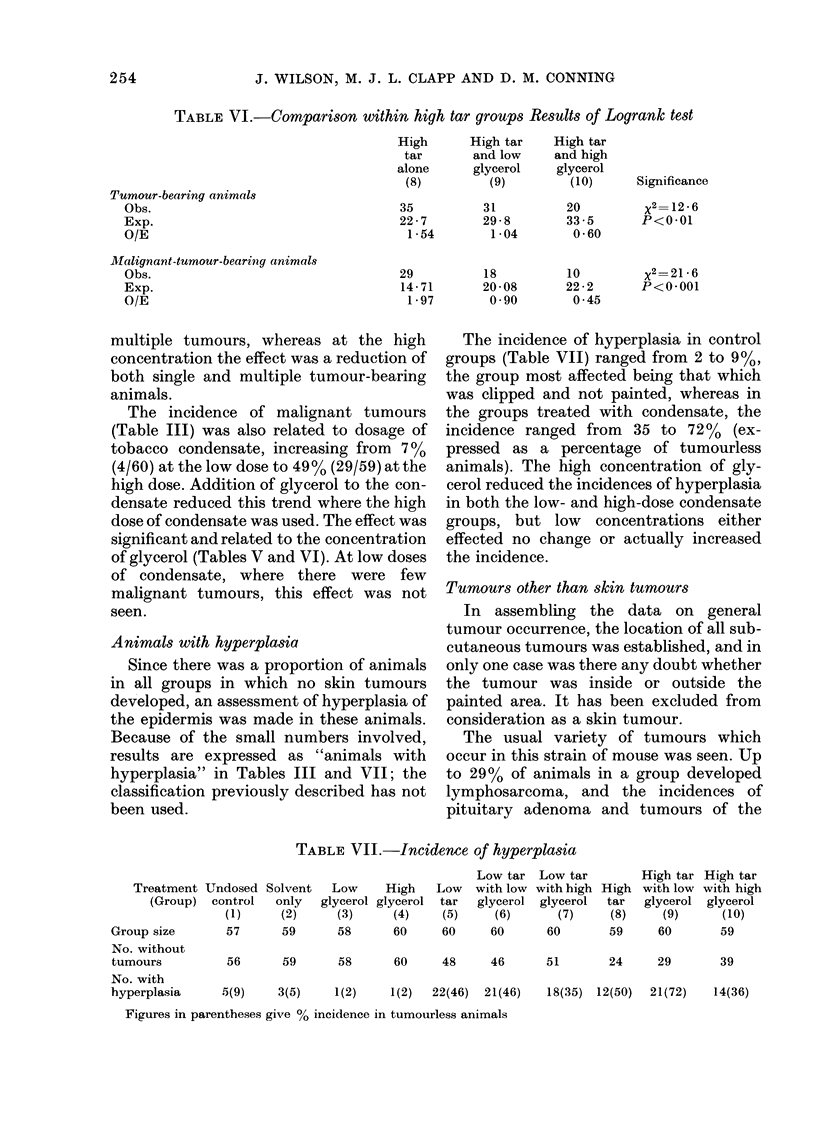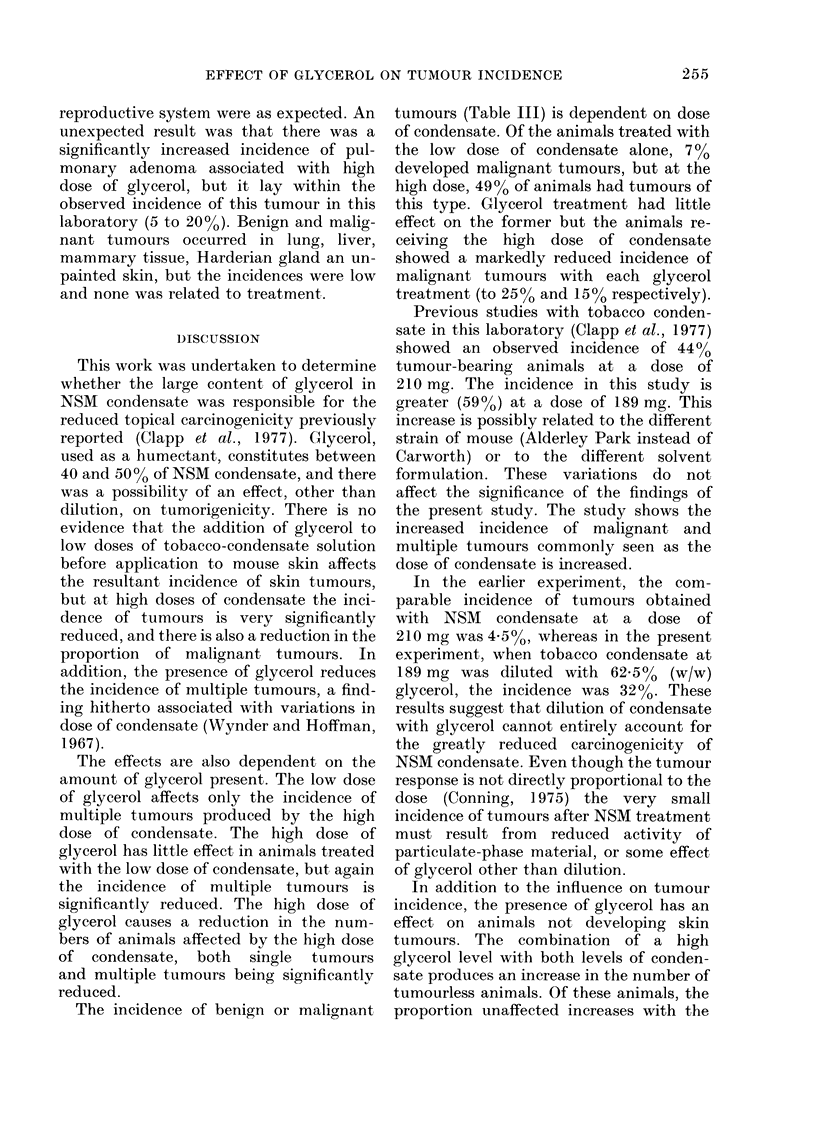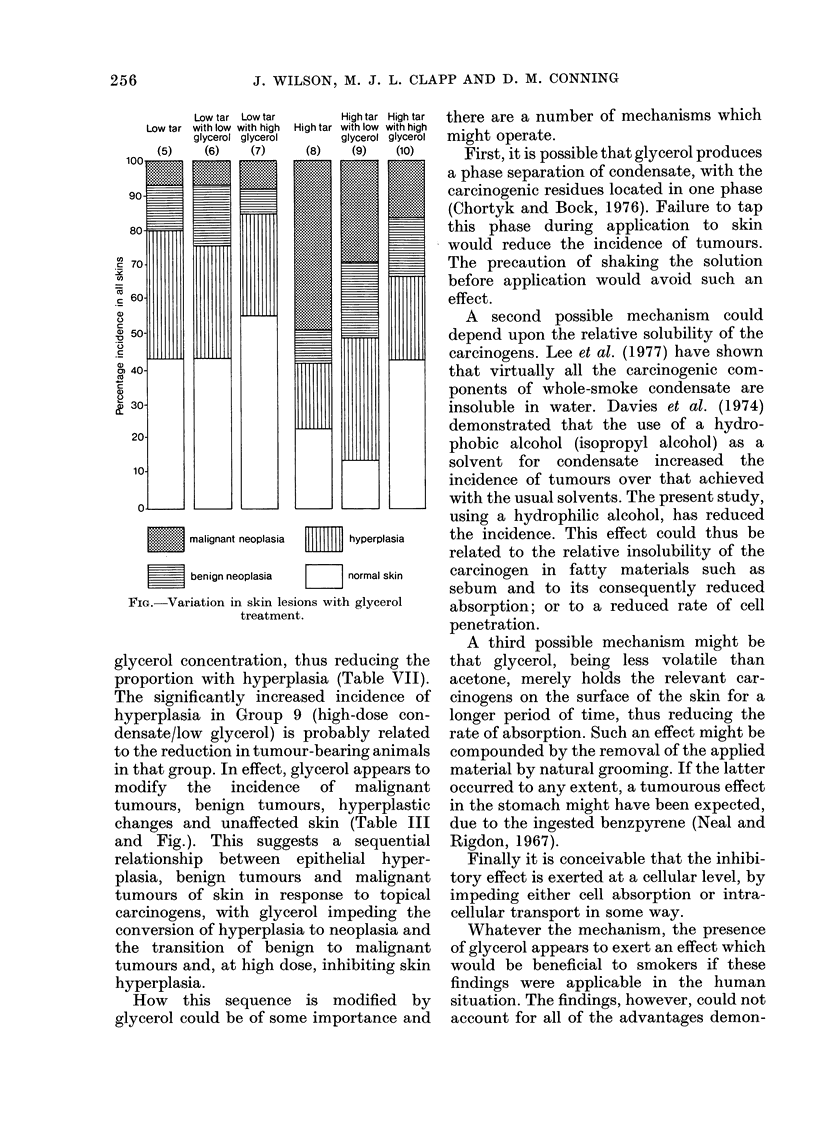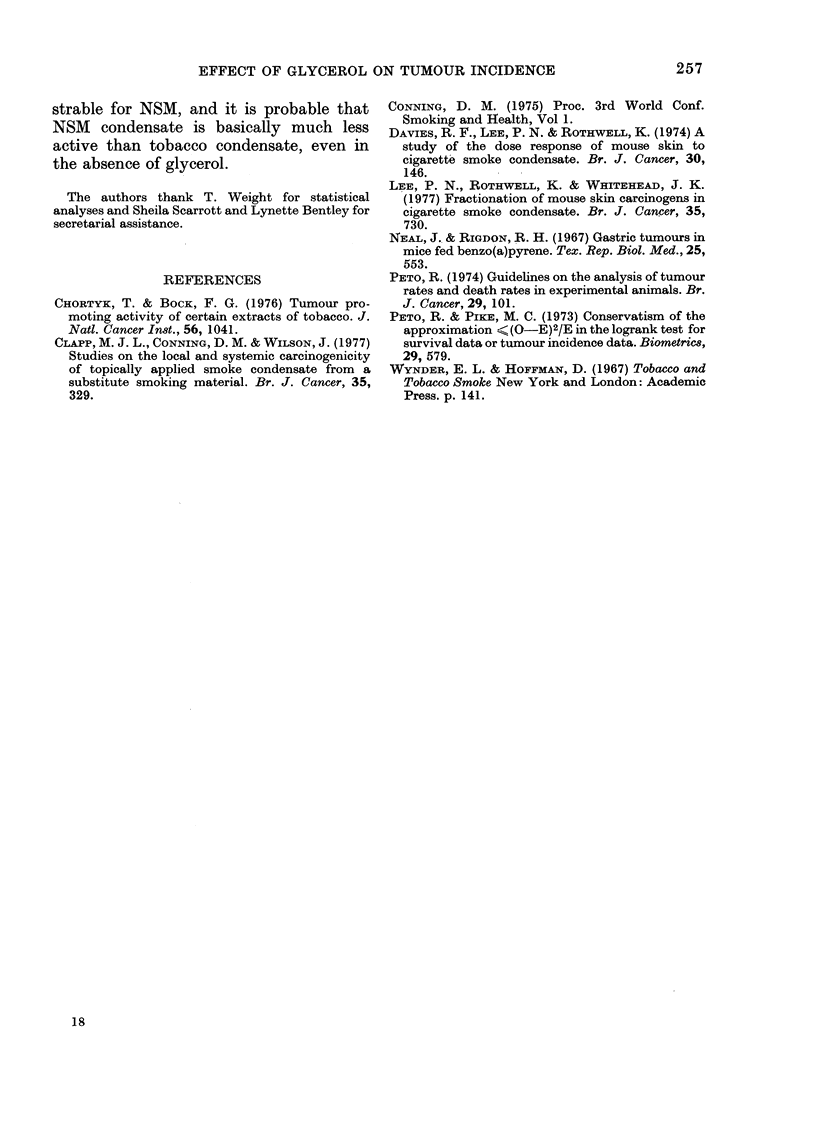# Effect of glycerol on local and systemic carcinogenicity of topically applied tobacco condensate.

**DOI:** 10.1038/bjc.1978.195

**Published:** 1978-08

**Authors:** J. Wilson, M. J. Clapp, D. M. Conning

## Abstract

When glycerol was added to tobacco smoke condensate in acetone solvent, the topical carcinogenicity and the ability to produce epithelial hyperplasia in mice was reduced. Two doses of condensate were applied, combined with 2 concentrations of added glycerol. Age-standardized results show that glycerol reduced the incidence of tumours and malignant tumours and of hyperplasia in animals not developing skin tumours. The relative incidences of malignant tumours, benign tumours, hyperplasia and unaffected skin suggest that there is a sequential relationship (i.e. normal skin to hyperplasia to benign neoplasia to malignant neoplasia) which is impeded by glycerol. There was no systemic effect attributable to the condensate.


*Br. J. Cancer* (1978) **38**, 250

### EFFECT OF GLYCEROL ON LOCAL AND SYSTEMIC CARCINOGENICITY OF TOPICALLY APPLIED TOBACCO CONDENSATE

J. WILSON, M. J. L. CLAPP AND D. M. CONNING†

*From the Central Toxicology Laboratory, Imperial Chemical Industries Ltd, Alderley Park, Macclesfield, Cheshire SK10 4TJ*

Received 3 February 1978 Accepted 8 May 1978

**Summary.—When glycerol was added to tobacco smoke condensate in acetone solvent, the topical carcinogenicity and the ability to produce epithelial hyperplasia in mice was reduced. Two doses of condensate were applied, combined with 2 concentrations of added glycerol. Age-standardized results show that glycerol reduced the incidence of tumours and malignant tumours and of hyperplasia in animals not developing skin tumours. The relative incidences of malignant tumours, benign tumours, hyperplasia and unaffected skin suggest that there is a sequential relationship (i.e. normal skin to hyperplasia to benign neoplasia to malignant neoplasia) which is impeded by glycerol. There was no systemic effect attributable to the condensate.**

IN STUDIES on the carcinogenicity of smoke condensate of tobacco and NSM* it was noted that NSM condensates showed less than 25% of the tumour-producing activity of tobacco (Clapp *et al.*, 1977). This was attributed to the general reduction of the particulate-phase activity of NSM smoke, but comment was made that the substantial carry-over into smoke of the humectant used (glycerol) might have been a factor of significance. Glycerol constitutes between 40% and 50% of the particulate phase of NSM smoke and it seemed possible that this amount might influence the response of mouse skin to the carcinogenic effect of smoke condensate, quite apart from any effect of the dilution of carcinogenic constituents. The experiment described here was designed to determine whether the presence of glycerol, as a diluent of tobacco condensate, itself influenced the carcinogenicity of the condensate irrespective of an effect on dosage.

### MATERIALS AND METHODS

*Cigarettes.*—The cigarettes from which condensate was prepared were 70 mm long and 25·4 mm in circumference. They were made from a commercial blend of flue-cured tobacco and contained no crushed stem. They were supplied by Imperial Tobacco Limited, Bristol.

*Glycerol.*—The glycerol was "Analar" reagent grade supplied by British Drug Houses in a single batch. It was stored at room temperature.

*Condensate preparation and application.*—The method was as previously reported (Clapp *et al.*, 1977), except that the acetone:water solvent was used in the proportion of 80:20 v/v rather than the conventional 90:10 v/v in order to minimize phase separation in those condensate preparations which contained high concentrations of glycerol.

Condensates were prepared at weekly intervals, stored at room temperature and applied to the animals with an ARH continuous pipetting unit (Arnold R. Horwell Limited) through a stainless-steel cannula, 4 cm long and 2 mm diameter. The dose applied was 0·3 ml per mouse, on 3 days each week (Monday, Wednesday and Friday). All condensates were shaken immediately before painting each cageful of mice, and a fixed volume was applied to each animal, different doses being achieved by use of different concentrations. Undosed control animals were

* Trade mark of New Smoking Materials Ltd.
† Present address: B.I.B.R.A., Woodmansterne Road, Carshalton, Surrey.

handled and shaved but not painted; solvent-control animals were painted with equivalent volumes of solvent or solvent/glycerol mixtures.

*Animals and treatment.*—A total of 600 female specific-pathogen-free mice of an Alderley Park albino strain, hysterectomy-derived of Swiss origin (ICI Limited, Pharmaceuticals Division, Alderley Park, Cheshire) were used.

The mice were housed 10 per cage, fed pasteurized cubed mouse diet (Oakes of Congleton, Cheshire) and tap water *ad libitum.* The cages measured $28 \cdot 5 \times 28 \cdot 0 \times$ 10 cm, were made from 19-guage galvanized-wire mesh and were suspended over collecting trays lined with absorbent paper. The animals were housed in one rack, and its position within the animal room was changed at intervals to avoid environmental effects of ventilation, light or temperature fluctuations. The animals were kept within a barriered-maintained area throughout the experiment, with the temperature maintained at about $22 \pm 1$°C and the relative humidity at about $55 \pm 10\%$.

After one week, mice were allocated to the different groups on the basis of total body weight per cage so as to give equal weight distribution between the groups. Each was clipped from the base of the tail to the nape of the neck with an Oster A5 electric hair clipper with a size-40 blade lubricated with liquid paraffin. This was repeated weekly throughout the study. Particular care was taken to avoid lacerating the skin, especially when tumours developed. A vacuum attachment over the oscillating teeth reduced the dispersion of contaminated hair into the room atmosphere. Sometimes, in the early stages of the experiment, the hair needed cleaning with acetone to remove inspissated tar, before clipping. Unpainted controls were clipped once a week. Treatment after 2 weeks, when mice were 6–7 weeks old and continued until death, or until 108 weeks of treatment had been completed.

All the experimental data were recorded on cards (Copeland-Chattersen Company Ltd) so that one card represented the history of one mouse.

*Experimental design.*—The experiment was designed to measure the effect of added glycerol on the carcinogenicity of tobacco condensate. The doses of condensate used were 189 and 94·5 mg/mouse/week, each alone or with 17·5% or 35% glycerol, the balance being made up from solvent (Table I). These doses were chosen to avoid high-dose suppression and thus to give optimum tumour response (Davies *et al.*, 1974), and to be comparable with glycerol proportions in NSM smoke condensate. Control groups received acetone/glycerol mixtures, or were handled and not otherwise treated (Table I).

All animals were checked daily and any abnormality in behaviour or physical condition recorded. Once a week, special attention was given to the skin, and a count and initial clinical classification of any tumours present was made; the exact position and date of appearance were noted on the mouse's record card.

Papillomas and suspected sebaceous adenomas were recorded when they appeared to be greater than 1 mm$^3$ and had been present on 2 consecutive weekly inspections. Papillomas were recorded as suspected carcinomas when there was swelling of the tissues beneath the papilloma, or when it became "fixed" to underlying tissues and a connection was suspected; or when sloughing, ulceration or "rolled edges" were seen. Regression was recorded when a papilloma completely disappeared. These observations were made on all animals. During the experiment, operator bias was minimized by ensuring that the same operator made decisions across all groups.

A full postmortem examination was made on all animals. Any animal which became distressed or moribund was killed and examined. Mice which died were examined within 24 h of death. Where autolysis was severe, skin alone was fixed for histological examination. In 2·5% of control animals (Groups 1–4) and 0·5% of test animals (Groups 5–10) all tissues were lost because of autolysis or cannibalism.

The following tissues were preserved in formol-corrosive for subsequent histopathological examination: adrenal, bladder, heart, stomach, duodenum, jejunum, ileum, caecum, colon, lungs (after inflation with formol saline), kidney, liver, mammary tissue, ovary, pancreas, pituitary, salivary glands, spleen, thymus (where identifiable), thyroid, uterus and voluntary muscle. Lymph nodes were taken where abnormal, or if they were draining an area with tumour. Grossly abnormal tissues extra to those specified were also taken.

The painted area of the skin, and all skin tumours inside and outside this area, were

TABLE I.—*Experimental design (All groups of* 60 *mice)*

| Group | Treatment | Tobacco tar mg/week | Glycerol mg/week |
|---|---|---|---|
| 1 | Undosed control | 0 | 0 |
| 2 | Solvent control | 0 | 0 |
| 3 | Low glycerol in solvent | 0 | 157·5 |
| 4 | High glycerol in solvent | 0 | 315 |
| 5 | Low tobacco tar | 94·5 | 0 |
| 6 | Low tobacco tar with low glycerol | 94·5 | 157·5 |
| 7 | Low tobacco tar with high glycerol | 94·5 | 315 |
| 8 | High tobacco tar | 189 | 0 |
| 9 | High tobacco tar with low glycerol | 189 | 157·5 |
| 10 | High tobacco tar with high glycerol | 189 | 315 |

preserved in Bouin's fixative. Each sample included some normal skin.

Tissues were examined microscopically for the presence of tumours. Hyperplasia was assessed in skin samples where tumours were not seen. Three pathologists examined the slides, which were distributed so that each received material from mice in all experimental groups. Diagnoses were coded for computer storage.

Analysis of hyperplasia and tumour diagnosis was based on criteria previously reported (Clapp *et al.*, 1977).

*Statistical methods.*—Differences in mortality between groups were compared by the Logrank test (Peto and Pike, 1973). This method was also used for the analysis of skin-tumour incidence and of the incidence of hyperplasia. For skin tumours the method used the time of first appearance of the tumour. Hyperplasia was treated as if it were an incidental finding (Peto, 1974) and animals with skin tumours were omitted. Both analyses allow for differing mortality rates. The skin-tumour analysis was done for tumour-bearing animals and for malignant-tumour-bearing animals.

### RESULTS

The general condition of the mice remained good throughout the study, with no evidence of intercurrent infection. Animals given the high doses of tobacco condensate showed signs of nicotine poisoning during the first few weeks, but thereafter tolerated the dose.

There was some evidence of differences in mortality between groups (Table II). This was largely due to a higher mortality in animals dosed with the high level of tobacco condensate alone.

### *Skin tumours*

The total number of tumours in the painted area (Table III) includes connective-tissue tumours beneath the painted area. All tumours were confirmed histo-

TABLE II.—*Cumulative mortality data*

| Treatment (Group) / Weeks on test | Untreated control (1) | Solvent control (2) | Low glycerol and solvent (3) | High glycerol and solvent (4) | Low tobacco (5) | Low tobacco and low glycerol (6) | Low tobacco and high glycerol (7) | High tobacco (8) | High tobacco and low glycerol (9) | High tobacco and high glycerol (10) |
|---|---|---|---|---|---|---|---|---|---|---|
| 12 | 1 | 0 | 0 | 0 | 0 | 0 | 1 | 0 | 0 | 0 |
| 24 | 4 | 1 | 1 | 0 | 1 | 0 | 2 | 1 | 1 | 0 |
| 36 | 5 | 3 | 1 | 2 | 1 | 2 | 2 | 3 | 1 | 1 |
| 48 | 10 | 5 | 3 | 5 | 2 | 5 | 5 | 6 | 3 | 5 |
| 60 | 18 | 9 | 9 | 9 | 6 | 13 | 8 | 9 | 4 | 9 |
| 72 | 21 | 11 | 13 | 15 | 14 | 20 | 13 | 17 | 16 | 14 |
| 84 | 27 | 21 | 29 | 27 | 20 | 30 | 24 | 30 | 31 | 23 |
| 96 | 36 | 33 | 38 | 40 | 36 | 41 | 28 | 45 | 41 | 42 |
| 108 | 47 | 45 | 47 | 49 | 50 | 50 | 49 | 60 | 51 | 53 |

Some variation between groups. No relationship overall with tobacco or glycerol. The highest mortality was in Group 8 but this was not evident until Week 96.

TABLE III.—*Incidence of skin lesions*

| Treatment (Group) | Low tar (5) | Low tar and low glycerol (6) | Low tar and high glycerol (7) | High tar (8) | High tar and low glycerol (9) | High tar and high glycerol (10) |
|---|---|---|---|---|---|---|
| Group size | 60 | 60 | 60 | 59 | 60 | 59 |
| Animals with normal skins | 26 | 26 | 33 | 13 | 8 | 25 |
| Animals with hyperplastic skins alone | 22 | 20 | 18 | 11 | 21 | 14 |
| Animals with benign skins tumours alone | 8 | 10 | 4 | 6 | 13 | 10 |
| Animals with malignant skin tumours alone | 4 | 4 | 5 | 29 | 18 | 10 |
| Total animals with skin tumours | 12 | 14 | 9 | 35 | 31 | 20 |
| Animals with a single skin tumour | 7 | 8 | 8 | 18+ | 21+ | 12+ |
| Animals with >1 skin tumour | 5 | 6+ | 1 | 17 | 10++ | 8 |

+ one animal with fibrosarcoma
++ two animals with fibrosarcoma

TABLE IV.—*Comparison within groups without glycerol treatment; Results of Logrank test*

| | Low tar (5) | High tar (8) | Significance |
|---|---|---|---|
| *Tumour-bearing animals* | | | |
| Observed no. | 12 | 35 | $\chi^2=29\cdot1$ |
| Expected no. | 29·83 | 17·17 | $P<0\cdot001$ |
| O/E | 0·40 | 2·04 | |
| *Malignant-tumour-bearing animals* | | | |
| Observed no. | 4 | 29 | $\chi^2=31\cdot5$ |
| Expected no. | 20·02 | 12·98 | $P<0\cdot001$ |
| O/E | 0·20 | 2·23 | |

logically. Only one tumour was seen in a control animal (Groups 1–4). It occurred in an undosed animal and proved to be a sebaceous adenoma. It was first noted at Week 80.

There was a significant dose-related tumour response in animals treated with tobacco condensate alone; the incidence was 20% (12/60) at the low dose and 59% (35/59) at the high dose. In these tumour-bearing animals there was an approximately equal incidence of single and multiple tumours.

Treatment with glycerol affected the tumour incidence (Tables III, V, VI). At the low dose of condensate, the high concentration of glycerol reduced tumour incidence by an effect on the number of animals with multiple tumours. The incidence of single-tumour-bearers was not changed. Among animals treated with a high dose of condensate, both concentrations of glycerol reduced the tumour incidence, the effect at low concentration being on the number of animals with

TABLE V.—*Comparison within low-tar groups; Results of Logrank test*

| | Low tar alone (5) | Low tar and low glycerol (6) | Low tar and high glycerol (7) | Significance |
|---|---|---|---|---|
| *Tumour-bearing animals* | | | | |
| Obs. | 12 | 14 | 9 | $\chi^2=2\cdot8$ |
| Exp. | 11·7 | 10·2 | 13·1 | (not significant) |
| O/E | 1·03 | 1·37 | 0·69 | |
| *Malignant tumour-bearing animals* | | | | |
| Obs. | 4 | 4 | 5 | |
| Exp. | 4·2 | 3·8 | 5·1 | $\chi^2=0\cdot03$ |
| O/E | 0·96 | 1·07 | 0·98 | (not significant) |

TABLE VI.—*Comparison within high tar groups Results of Logrank test*

| | High tar alone (8) | High tar and low glycerol (9) | High tar and high glycerol (10) | Significance |
|---|---|---|---|---|
| *Tumour-bearing animals* | | | | |
| Obs. | 35 | 31 | 20 | $\chi^2 = 12 \cdot 6$ |
| Exp. | 22·7 | 29·8 | 33·5 | $P < 0 \cdot 01$ |
| O/E | 1·54 | 1·04 | 0·60 | |
| *Malignant-tumour-bearing animals* | | | | |
| Obs. | 29 | 18 | 10 | $\chi^2 = 21 \cdot 6$ |
| Exp. | 14·71 | 20·08 | 22·2 | $P < 0 \cdot 001$ |
| O/E | 1·97 | 0·90 | 0·45 | |

multiple tumours, whereas at the high concentration the effect was a reduction of both single and multiple tumour-bearing animals.

The incidence of malignant tumours (Table III) was also related to dosage of tobacco condensate, increasing from 7% (4/60) at the low dose to 49% (29/59) at the high dose. Addition of glycerol to the condensate reduced this trend where the high dose of condensate was used. The effect was significant and related to the concentration of glycerol (Tables V and VI). At low doses of condensate, where there were few malignant tumours, this effect was not seen.

### *Animals with hyperplasia*

Since there was a proportion of animals in all groups in which no skin tumours developed, an assessment of hyperplasia of the epidermis was made in these animals. Because of the small numbers involved, results are expressed as "animals with hyperplasia" in Tables III and VII; the classification previously described has not been used.

The incidence of hyperplasia in control groups (Table VII) ranged from 2 to 9%, the group most affected being that which was clipped and not painted, whereas in the groups treated with condensate, the incidence ranged from 35 to 72% (expressed as a percentage of tumourless animals). The high concentration of glycerol reduced the incidences of hyperplasia in both the low- and high-dose condensate groups, but low concentrations either effected no change or actually increased the incidence.

### *Tumours other than skin tumours*

In assembling the data on general tumour occurrence, the location of all subcutaneous tumours was established, and in only one case was there any doubt whether the tumour was inside or outside the painted area. It has been excluded from consideration as a skin tumour.

The usual variety of tumours which occur in this strain of mouse was seen. Up to 29% of animals in a group developed lymphosarcoma, and the incidences of pituitary adenoma and tumours of the

TABLE VII.—*Incidence of hyperplasia*

| Treatment (Group) | Undosed control (1) | Solvent only (2) | Low glycerol (3) | High glycerol (4) | Low tar (5) | Low tar with low glycerol (6) | Low tar with high glycerol (7) | High tar (8) | High tar with low glycerol (9) | High tar with high glycerol (10) |
|---|---|---|---|---|---|---|---|---|---|---|
| Group size | 57 | 59 | 58 | 60 | 60 | 60 | 60 | 59 | 60 | 59 |
| No. without tumours | 56 | 59 | 58 | 60 | 48 | 46 | 51 | 24 | 29 | 39 |
| No. with hyperplasia | 5(9) | 3(5) | 1(2) | 1(2) | 22(46) | 21(46) | 18(35) | 12(50) | 21(72) | 14(36) |

Figures in parentheses give % incidence in tumourless animals reproductive system were as expected. An unexpected result was that there was a significantly increased incidence of pulmonary adenoma associated with high dose of glycerol, but it lay within the observed incidence of this tumour in this laboratory (5 to 20%). Benign and malignant tumours occurred in lung, liver, mammary tissue, Harderian gland an unpainted skin, but the incidences were low and none was related to treatment.

### DISCUSSION

This work was undertaken to determine whether the large content of glycerol in NSM condensate was responsible for the reduced topical carcinogenicity previously reported (Clapp *et al.*, 1977). Glycerol, used as a humectant, constitutes between 40 and 50% of NSM condensate, and there was a possibility of an effect, other than dilution, on tumorigenicity. There is no evidence that the addition of glycerol to low doses of tobacco-condensate solution before application to mouse skin affects the resultant incidence of skin tumours, but at high doses of condensate the incidence of tumours is very significantly reduced, and there is also a reduction in the proportion of malignant tumours. In addition, the presence of glycerol reduces the incidence of multiple tumours, a finding hitherto associated with variations in dose of condensate (Wynder and Hoffman, 1967).

The effects are also dependent on the amount of glycerol present. The low dose of glycerol affects only the incidence of multiple tumours produced by the high dose of condensate. The high dose of glycerol has little effect in animals treated with the low dose of condensate, but again the incidence of multiple tumours is significantly reduced. The high dose of glycerol causes a reduction in the numbers of animals affected by the high dose of condensate, both single tumours and multiple tumours being significantly reduced.

The incidence of benign or malignant tumours (Table III) is dependent on dose of condensate. Of the animals treated with the low dose of condensate alone, 7% developed malignant tumours, but at the high dose, 49% of animals had tumours of this type. Glycerol treatment had little effect on the former but the animals receiving the high dose of condensate showed a markedly reduced incidence of malignant tumours with each glycerol treatment (to 25% and 15% respectively).

Previous studies with tobacco condensate in this laboratory (Clapp *et al.*, 1977) showed an observed incidence of 44% tumour-bearing animals at a dose of 210 mg. The incidence in this study is greater (59%) at a dose of 189 mg. This increase is possibly related to the different strain of mouse (Alderley Park instead of Carworth) or to the different solvent formulation. These variations do not affect the significance of the findings of the present study. The study shows the increased incidence of malignant and multiple tumours commonly seen as the dose of condensate is increased.

In the earlier experiment, the comparable incidence of tumours obtained with NSM condensate at a dose of 210 mg was 4·5%, whereas in the present experiment, when tobacco condensate at 189 mg was diluted with 62·5% (w/w) glycerol, the incidence was 32%. These results suggest that dilution of condensate with glycerol cannot entirely account for the greatly reduced carcinogenicity of NSM condensate. Even though the tumour response is not directly proportional to the dose (Conning, 1975) the very small incidence of tumours after NSM treatment must result from reduced activity of particulate-phase material, or some effect of glycerol other than dilution.

In addition to the influence on tumour incidence, the presence of glycerol has an effect on animals not developing skin tumours. The combination of a high glycerol level with both levels of condensate produces an increase in the number of tumourless animals. Of these animals, the proportion unaffected increases with the

FIG.—Variation in skin lesions with glycerol treatment.

glycerol concentration, thus reducing the proportion with hyperplasia (Table VII). The significantly increased incidence of hyperplasia in Group 9 (high-dose condensate/low glycerol) is probably related to the reduction in tumour-bearing animals in that group. In effect, glycerol appears to modify the incidence of malignant tumours, benign tumours, hyperplastic changes and unaffected skin (Table III and Fig.). This suggests a sequential relationship between epithelial hyperplasia, benign tumours and malignant tumours of skin in response to topical carcinogens, with glycerol impeding the conversion of hyperplasia to neoplasia and the transition of benign to malignant tumours and, at high dose, inhibiting skin hyperplasia.

How this sequence is modified by glycerol could be of some importance and there are a number of mechanisms which might operate.

First, it is possible that glycerol produces a phase separation of condensate, with the carcinogenic residues located in one phase (Chortyk and Bock, 1976). Failure to tap this phase during application to skin would reduce the incidence of tumours. The precaution of shaking the solution before application would avoid such an effect.

A second possible mechanism could depend upon the relative solubility of the carcinogens. Lee *et al.* (1977) have shown that virtually all the carcinogenic components of whole-smoke condensate are insoluble in water. Davies *et al.* (1974) demonstrated that the use of a hydrophobic alcohol (isopropyl alcohol) as a solvent for condensate increased the incidence of tumours over that achieved with the usual solvents. The present study, using a hydrophilic alcohol, has reduced the incidence. This effect could thus be related to the relative insolubility of the carcinogen in fatty materials such as sebum and to its consequently reduced absorption; or to a reduced rate of cell penetration.

A third possible mechanism might be that glycerol, being less volatile than acetone, merely holds the relevant carcinogens on the surface of the skin for a longer period of time, thus reducing the rate of absorption. Such an effect might be compounded by the removal of the applied material by natural grooming. If the latter occurred to any extent, a tumourous effect in the stomach might have been expected, due to the ingested benzpyrene (Neal and Rigdon, 1967).

Finally it is conceivable that the inhibitory effect is exerted at a cellular level, by impeding either cell absorption or intracellular transport in some way.

Whatever the mechanism, the presence of glycerol appears to exert an effect which would be beneficial to smokers if these findings were applicable in the human situation. The findings, however, could not account for all of the advantages demonstrable for NSM, and it is probable that NSM condensate is basically much less active than tobacco condensate, even in the absence of glycerol.

The authors thank T. Weight for statistical analyses and Sheila Scarrott and Lynette Bentley for secretarial assistance.

### REFERENCES

CHORTYK, T. & BOCK, F. G. (1976) Tumour promoting activity of certain extracts of tobacco. *J. Natl. Cancer Inst.*, **56**, 1041.

CLAPP, M. J. L., CONNING, D. M. & WILSON, J. (1977) Studies on the local and systemic carcinogenicity of topically applied smoke condensate from a substitute smoking material. *Br. J. Cancer*, **35**, 329.

CONNING, D. M. (1975) Proc. 3rd World Conf. Smoking and Health, Vol 1.

DAVIES, R. F., LEE, P. N. & ROTHWELL, K. (1974) A study of the dose response of mouse skin to cigarette smoke condensate. *Br. J. Cancer*, **30**, 146.

LEE, P. N., ROTHWELL, K. & WHITEHEAD, J. K. (1977) Fractionation of mouse skin carcinogens in cigarette smoke condensate. *Br. J. Cancer*, **35**, 730.

NEAL, J. & RIGDON, R. H. (1967) Gastric tumours in mice fed benzo(a)pyrene. *Tex. Rep. Biol. Med.*, **25**, 553.

PETO, R. (1974) Guidelines on the analysis of tumour rates and death rates in experimental animals. *Br. J. Cancer*, **29**, 101.

PETO, R. & PIKE, M. C. (1973) Conservatism of the approximation $\leqslant(O-E)^2/E$ in the logrank test for survival data or tumour incidence data. *Biometrics*, **29**, 579.

WYNDER, E. L. & HOFFMAN, D. (1967) *Tobacco and Tobacco Smoke* New York and London: Academic Press. p. 141.